# Synergistic Effect of Composite Nickel Phosphide Nanoparticles and Carbon Fiber on the Enhancement of Salivary Enzyme-Free Glucose Sensing

**DOI:** 10.3390/bios13010049

**Published:** 2022-12-29

**Authors:** Tania P. Brito, Nicole Butto-Miranda, Andrónico Neira-Carrillo, Soledad Bollo, Domingo Ruíz-León

**Affiliations:** 1 Centro de Investigación de Procesos Redox (CiPRex), Facultad de Ciencias Químicas y Farmacéuticas, Universidad de Chile, Santiago 8330015, Chile; 2 Laboratorio de Fisicoquímica y Electroquímica del Estado Sólido, Facultad de Química y Biología, Universidad de Santiago de Chile, Santiago 8330015, Chile; 3 Departamento de Ingeniería Metalúrgica, Facultad de Ingeniería, Universidad de Santiago de Chile, Santiago 8330015, Chile; 4 Departamento de Ciencias Biológicas Animales, Facultad de Ciencias Veterinarias y Pecuarias, Universidad de Chile, Santiago 8330015, Chile

**Keywords:** nickel phosphide, enzyme-free glucose sensors, electrochemical sensors, salivary sensor

## Abstract

An electrospinning method was used for the preparation of an in situ composite based on Ni_2_P nanoparticles and carbon fiber (FC). The material was tested for the first time against direct glucose oxidation reaction. The Ni_2_P nanoparticles were distributed homogeneously throughout the carbon fibers with a composition determined by thermogravimetric analysis (TGA) of 40 wt% Ni_2_P and 60 wt% carbon fiber without impurities in the sample. The electrochemical measurement results indicate that the GCE/FC/Ni_2_P in situ sensor exhibits excellent catalytic activity compared to the GCE/Ni_2_P and GCE/FC/Ni_2_P ex situ electrodes. The GCE/FC/Ni_2_P in situ sensor presents a sensitivity of 1050 µAmM^−1^cm^−2^ in the range of 5–208 µM and a detection limit of 0.25 µM. The sensor was applied for glucose detection in artificial saliva, with a low interference observed from normally coexisting electroactive species. In conclusion, our sensor represents a novel and analytical competitive alternative for the development of non-enzymatic glucose sensors in the future.

## 1. Introduction

The measurement of glucose through reliable methods is a very relevant issue due to its increased demand for medical diagnoses of diabetes mellitus (DM). Thus, its early detection will improve the quality of life in patients, considering that DM leads to many deaths worldwide [1]. Many sensors have been developed to monitor glucose levels in patients with DM. Currently, commercial electrochemical glucose sensors are invasive, due to the need for a blood sample, and are based on enzymatic proteins as recognition elements, with glucose oxidase being the most commonly used. Despite their high sensitivity and selectivity, these sensors have a high cost and are unstable with changes in temperature, pH, and humidity due to the enzymes used, making their practical applications difficult [2,3]. To avoid these drawbacks, it is of great importance to develop sensitive and selective non-enzymatic sensors for glucose detection in less painful or non-invasive matrixes. The non-enzymatic glucose sensors have emerged as a new development alternative. Their performance, together with that of enzymatic biosensors, has been recently reviewed by Nor et al., remarking that they “have an excellent sensitivity, good stability and ease of manufacture, and their current response is directly dependent on the oxidation of glucose on the modified electrode” [3].

Noble metals and their metal alloys possess high catalytic activity for the non-enzymatic electrooxidation of glucose, but the high cost of such catalysts limits their widespread applications [4]. Alternatively, the use of abundant elements on earth, such as some transition metals and their oxides and hydroxides, is a very attractive option for electrocatalytic glucose detection. However, despite being cheaper, the widespread use of metal oxide-based sensors is hampered mainly by their low electrical conductivity. To avoid this problem, hybrid materials based on inorganic salts coupled to conductive substrates such as graphene, nanotubes, and carbon nanofibers have been designed [5,6,7,8]. As a result, these materials acquire a greater surface area and a more effective electron transfer. Another strategy is based on the search for ceramic materials with higher intrinsic electrical conductivity, such as transition metal phosphides. Recently, these compounds have drawn attention due to their excellent chemical and physical stability, as well as their great catalytic activity, comparable to that of noble metals, in different applications [9,10,11]. However, the study of these compounds in the area of non-enzymatic sensors has been explored less.

Nickel is an attractive transition metal for the electrooxidation of glucose with high catalytic activities. The efficacy of glucose sensors based on Ni oxides and hydroxides has been extensively demonstrated in the literature [12,13,14,15]. In general terms, the use of these Ni oxides involves potentials closer to 0.6 V with reported detection limits in the range of 5 to 20 micromolar, even when combined with carbon nanomaterials. In the last few years, nickel phosphide (Ni_2_P) has emerged as a new material due to its abundance in the earth’s crust, excellent chemical stability, good electrical conductivity, and high catalytic activity. It has been used as catalysts in the reactions of hydrodesulfurization [16], hydrodenitrogenation [16], and evolution of hydrogen [9,17,18,19], where catalytic activities have been obtained close to that of noble metals [17]. Moreover, Ni_2_P has been applied in photocatalytic degradation, and in lithium-ion batteries [20]. Regarding glucose sensors based on Ni_2_P specifically, there are only three reports in the literature with two involving carbon materials: Ni_2_P nanosheets supported on carbon cloth, Ni_2_P NA/CC [21] and Ni_2_P(MOF)/Graphene [22]. The third one corresponds to Ni_2_P nanosheets on a nickel foam, Ni_2_P/NF [23]. All of them show good sensitivities and a low limit of detection and were applied for monitoring blood glucose.

In this work, the electrospinning technique followed by heat treatment in an Ar/H_2_ atmosphere was applied to manufacture a hybrid material based on the joint synthesis of Ni_2_P with carbon fiber (FC/Ni_2_P), called in situ, to prepare a low cost new composite which has not been reported until now for glucose sensing. The in situ Ni_2_P/FC composite was fully characterized and applied as electrocatalytic material for salivary non-invasive diabetes monitoring. For comparison, carbon fiber and Ni_2_P were synthesized individually. Using these individual compounds, the effectiveness of the electrocatalytic materials of carbon fiber, Ni_2_P, and ex situ and in situ Ni_2_P/FC composites for glucose electrooxidation was compared.

## 2. Materials and Methods

### 2.1. Reagents

All chemicals were of analytical grade. Nickel chloride hexahydrate (NiCl_2_∙6H_2_O, Aldrich, St. Louis, MO, USA), nickel nitrate (Ni(NO_3_)_2_∙6H_2_O, Merck, Darmstadt, Alemania), nickel sulfate hexadritadate (NiSO_4_∙6H_2_O, Merck), sodium phosphinate monohydrate (NaPO_2_H_2_·H_2_O, Merck), polyvinyl porrolidone (C_6_H_9_NO)n, PVP, MW = 1,300,000), orthophosphoric acid (H_3_PO_4_ 85%, Merck), N,N dimethylformamide (C_3_H_7_NO, DMF Merck) hexamethylenediamine (C_6_H_16_N_2_, Merck), 0.3 and 0.05 µm aluminas (Buehler, Uzwil, Switzerland), α-D-Glucose (C_6_H_12_O_6_ Sigma-Aldrich), Nafion (5.0% Aldrich), Sodium Hydroxide (NaOH, Merck), Ethanol (Analytical Grade, Merck), Acetone (Analytical Grade, Merck), ethylenediamine (Merck). For the thermal treatments, H_2_ and Ar (Airliquide, Paris, France) were used. All electrochemical solutions were prepared with ultrapure water (ρ = 18.2 MΩ cm) from a Millipore-MilliQ system that was used for preparing all aqueous solutions.

### 2.2. Synthesis of Carbon Fiber (FC) and the FC/Ni_2_P In Situ Composite

The synthesis of carbon fiber and the in situ composite was carried out using electrospinning stretching LE-10 Fluidnatec equipment and 10 mL Nipro luer/lock syringes based on a modification work of Shu-Juan Bao et al. [18]. To prepare the electrospinning solution, polyvinylpyrrolidone (PVP) (10% *w*/*v* of the final 250 mL solution) was mixed in 125 mL of Milli-Q water and stirred. Then, 125 mL of N,N-dimethylformamide (DMF) was added, and the mixture was stirred. Once this time had elapsed, 50.0 mmol of 85% H_3_PO_4_ was added slowly, and the mixture was stirred at room temperature. A homogeneous solution was finally obtained. This solution was electrospun in the electrospinning equipment using a flow of 800 µL/h, applying a voltage of 18 KV and a distance between the needle and collector of 20 cm at room temperature with a relative humidity of 70%. The collection time was approximately 12 h. Once the polymer meshes were obtained they were dried at 110 °C for 12 h. Then, the stabilization process was carried out at 250 °C for 3 h in air using a ramp of 7 °C/min, and the samples were allowed to cool to room temperature. Subsequently, the obtained fibers were calcined at 700 °C for 3 h in Ar/H_2_ flow using a heating rate of 10 °C/min and cooled to room temperature in an Ar atmosphere. The fibers obtained were cooled to room temperature in an Ar atmosphere.

Like the synthesis of carbon fibers, the in situ Ni_2_P/FC composite was synthesized using the electrospinning technique. In this case, the precursor electrospinning solution was prepared from an aqueous solution of NiSO_4_ (50 mmol) in 125 mL of Milli-Q water, which was stirred. PVP (10% *w*/*v* of the final 125 mL solution) was then added and stirred again. Subsequently, 125 mL of N,N-dimethylformamide (DMF) was added and the mixture was stirred. Once this time had elapsed, 50 mmol of 85% H_3_PO_4_ was added slowly, and the mixture was stirred at room temperature. The homogeneous solution obtained was electrospun using the same parameters as for the carbon fiber. Once the polymer meshes were obtained, the same heat treatment was carried out as for the carbon fiber.

### 2.3. Synthesis of Ni_2_P

Following the work of Qingxin Guan et al. [24], Ni_2_P was prepared using nickel chloride (NiCl_2_) and sodium hypophosphite (NaPO_2_H_2_·H_2_O) as precursors, which were dissolved in Milli-Q water under stirring with an Ni:P = 1:1.5 molar ratio. After stirring for 1 h, the solution was slowly evaporated at 50 °C for 8 h and then left to stand at room temperature until the precursor was obtained. The precursor was dried at 90 °C and then ground in an agate mortar. The product obtained was treated at 300 °C for 30 min in an Ar atmosphere with a heating rate of 5 °C/min. The a was cooled to room temperature under flowing Ar and washed repeatedly with Milli-Q water and ethanol. Ni_2_P was finally obtained after drying at 120 °C for 6 h.

### 2.4. Materials Characterization

The crystalline structure and purity of the obtained materials were determined from X-ray diffraction (XRD). The X-ray diffraction data were collected at room temperature on a Bruker D8 Advance (R-X: Cu tube (CuKa1 radiation = 1.5604 angstroms), filter: nickel, detector: linear LynxEye, power used: 40 KV/30 mA, and optics: variable, V20). The spectra were measured in the 2θ range between 10 and 80° (PSD step = 0.20; 10.0 s/step). The analysis of crystalline phases was performed using the Crystal ImpactMatch program with the PDF-2 database. Parameter lattices were calculated using the STOE XPOW software. The surface morphology was studied using a Thermo Fisher Scientific (Waltham, MA, USA) INSPECT-F50 high-resolution scanning electron microscope (HR-SEM, FEI, Eindhoven, The Netherlands). The compositional study was conducted by energy dispersive spectroscopy (EDX) and backscattered electron (BSE) analysis, scanning transmission electron microscopy (STEM), and an Alpine 129 eV ultradry pathfinder (Thermo Fisher Scientific). The composition analysis of the in situ composite was carried out by thermogravimetric analysis using a TA Instruments Model Q50 thermobalance. The conditions of the experiment were a speed of 5 °C/min in air and a temperature of 30 °C to 800 °C.

### 2.5. Modification of Glassy Carbon Electrodes (GCEs) with the Materials

**Scheme 1:**
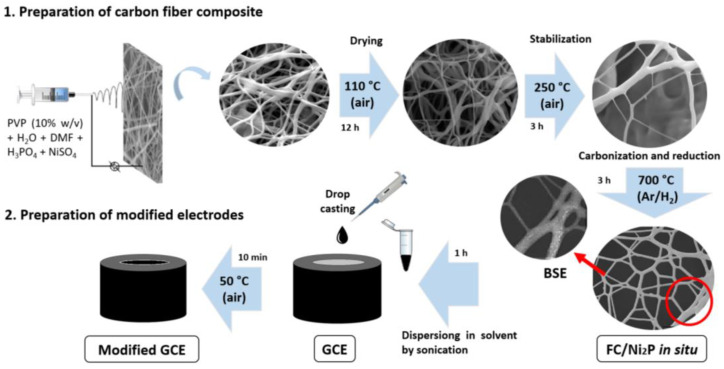
Preparation method for carbon fiber composite and modified glassy carbon electrode (GCE).

For the modification of glassy carbon electrodes, the drop casting technique was used. Dispersions of 2.0 mg/mL of the synthesized materials dissolved in 0.2% Nafion in isopropanol and water were prepared. For the case of the ex situ Ni_2_P/FC composite, the ratios were massed with 60 wt% FC, which corresponds to a concentration of 2.0 mg/mL. All dispersions were sonicated for 1 h. Before each modification, the glassy carbon electrode must be polished. The electrode was polished in 0.3 and 0.05 µm alumina and then washed with Milli-Q water. Ten microliters of the dispersion were taken and deposited on the surface of a glassy carbon electrode and then dried at 50 °C for 10 min. Scheme 1 illustrates the preparation, both of carbonized nanofiber composites and modified electrodes, successively.

### 2.6. Electrochemical Measurements

To explore the catalytic activity of the modified electrodes, a typical three-electrode system composed of a glassy carbon working electrode (CH Instrument, Bee Cave, TX, USA, 3 mm diameter), an Ag/AgCl reference electrode in 3 mol L^−1^ NaCl (CH Instrument), and an auxiliary platinum wire electrode was arranged in an electrochemical cell with a capacity of 10 mL in a MultiEmStat Potentiostat (Palmsens BV, Houten, The Netherlands). All electrochemical measurements were carried out in triplicate by immersing the modified electrode in 5 mL of 0.1 M NaOH as the supporting electrolyte.

The CV curves were measured at a scan rate of 50 mVs^−1^ for a potential range comprised between 0.200 and 0.700 V. The amperometric experiments were conducted at 0.500 V by applying the desired working potential and allowing the transient currents to decay to a steady-state value prior to the addition of glucose, with subsequent current monitoring. The practical application of the proposed sensor was carried out performed using artificial human saliva samples (SAGF medium) that was prepared as described by Gal et al. [25] and diluted in NaOH solution before measurements.

## 3. Results

### 3.1. Characterization of Materials

**Figure 1:**
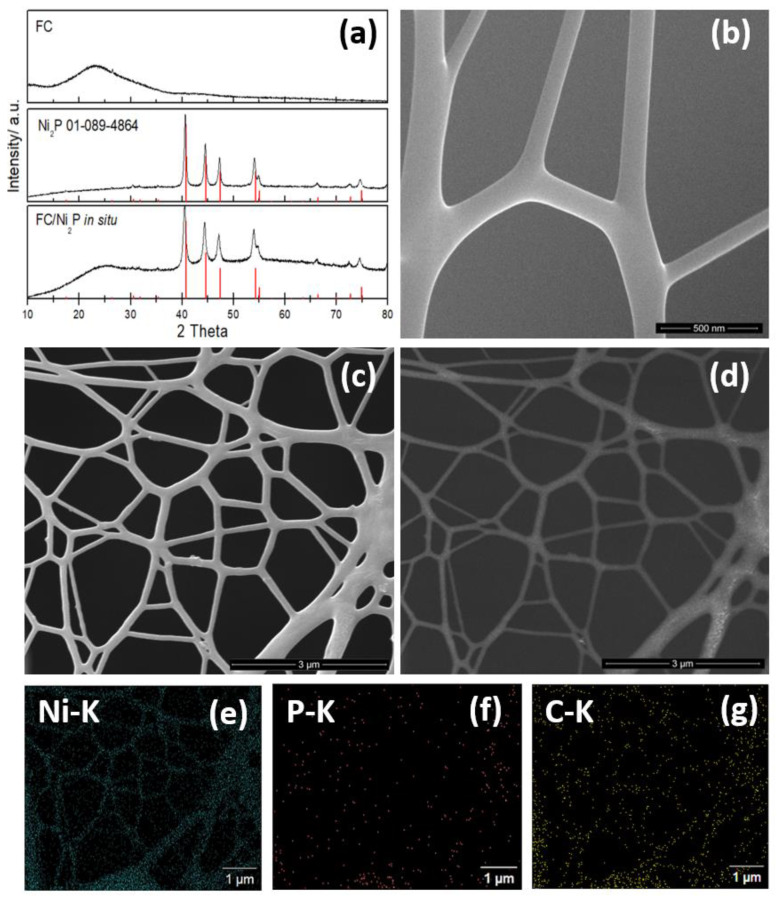
(**a**) X-ray diffraction of carbon fiber (FC), Ni_2_P and in situ FC/Ni_2_P composite. SEM images of (**b**) carbon fiber (FC) and (**c**) in situ FC/Ni_2_P composite. (**d**) Backscattering electron micrographs (BSE) of the in situ FC/Ni_2_P composite. Energy dispersive X-ray spectroscopy (EDX) of (**e**) nickel, (**f**) phosphorus and (**g**) carbon phosphorus.

The crystalline structure and purity of the obtained materials were determined from X-ray diffraction (XRD). Figure 1a shows the diffraction patterns of the carbon fiber (FC), Ni_2_P, and in situ Ni_2_P/FC composite. A diffraction peak at approximately 2θ = 24°, which is characteristic of carbon fibers, can be observed in the FC diffractograms [17]. Regarding Ni_2_P, the diffraction pattern agrees with that of Ni_2_P (PDF2 no. 01-089-4864), corresponding to space group P-62m with a hexagonal crystal system and cell parameters of a = 5.87 Å, c = 3.39 Å, and cell volume = 101.20 Å^3^. In the phase analysis of the in situ Ni_2_P/FC composite, it can be seen that the diffraction peaks correspond to the FC (2θ = 24°), and Ni_2_P (PDF2 no. 01-089-4864) diffraction planes. The Ni_2_P contained in the composite has a hexagonal crystal system with space group P-62m (189), whose cell parameters are a = 5.85 Å, b = 3.37 Å, and cell volume of 100.19 Å3. For all materials, no additional planes were observed that could be attributable to contaminants or unreacted precursors. Therefore, the proposed materials were successfully obtained from the electrospinning technique, and the thermal treatment used was effective. The Ni_2_P crystallite size of the individual compound and the in situ composite were obtained from the Debye Scherrer equation, corresponding to 17.13 ± 3.1 nm and 12.09 ± 1.5 nm, respectively. A very similar crystallite size was obtained in both materials. The particle size of individual Ni_2_P and that in the composite were studied from STEM images (Figure S1). From a Gaussian distribution analysis, an average particle size of 23.91 nm ± 1.9 nm for individual Ni_2_P and 29.10 ± 2.2 nm for Ni_2_P in the composite were determined.

Figure 1b,c shows the SEM images of the carbon fiber and in situ FC/Ni_2_P composite, respectively. Thus, it is possible to distinguish that the obtained fibers in both the carbon fiber and composite have a morphology of smooth threads, without pores or visible cracks. This fact implies that the stabilization and reduction treatments were carried out adequately, since these steps fulfill the function of slowly and gradually releasing volatile compounds, avoiding the formation of imperfections in the final carbon fiber [18]. The diameter of the fibers is practically the same in each step of the synthesis (Figures S2 and S3) with an average of 123 nm ± 54 nm in the bare carbon fibers and 118.0 nm ± 57 nm for the fibers in the composite, which could also be a consequence of the effective and gradual stabilization treatment.

**Figure 2:**
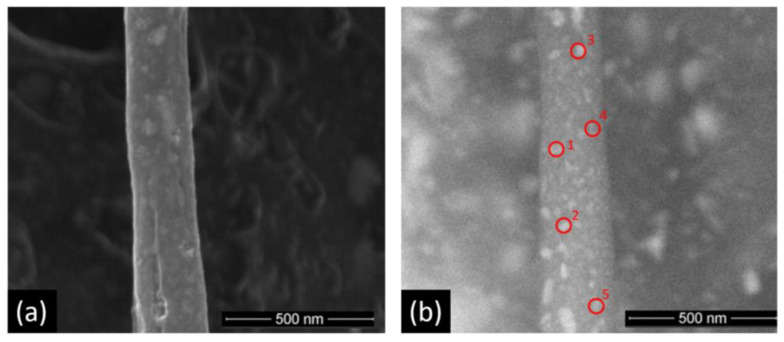
(**a**) SEM image of in situ FC/Ni_2_P composite, and (**b**) BSE image of in situ FC/Ni_2_P composite. An EDX analysis was performed per point from the points indicated in BSE image.

The distribution of Ni_2_P onto the carbon fiber is essential for electrocatalytic applications of sensors, and the composite is analyzed by back-scattered electron (BSE) microscopy and energy dispersive X-ray (EDX) elemental mapping analysis techniques. Figure 1d corresponds to the BSE images of the in situ FC/Ni_2_P composite. The observed bright spots indicate a homogeneous distribution of Ni_2_P all over the carbon fibers. The image is then mapped using EDX analysis, identifying nickel, phosphorus and carbon, in Figure 1e–g, respectively. Both Ni and P were identified and merged with the carbon matrix. On the other hand, from an EDX analysis per point, indicated in Figure 2, the average atomic percentage ratio between Ni:P was 2.13:1.0 (Figure S4 and Table S1), which is very close to the ratio 2.0:1.0 in the theoretical Ni_2_P. These analyses demonstrate the formation of Ni_2_P nanoparticles throughout the carbon fiber distributed homogeneously.

The determination of the composition of Ni_2_P and carbon fiber in the in situ composite was carried out employing thermogravimetric analysis (TGA) (Figure S5). The entire process initially includes the release of 8.3% by weight of water; then the decomposition of the organic matrix begins, which corresponds to 50.6% by weight. Based on the above, approximately 60 wt% carbon fiber is present, and the remaining 40 wt% is assigned to the inorganic material. This composition percentage was then used for the comparative electroanalytical tests to prepare the ex situ FC/Ni_2_P composite.

### 3.2. Electrocatalytic Performance toward Glucose

**Figure 3:**
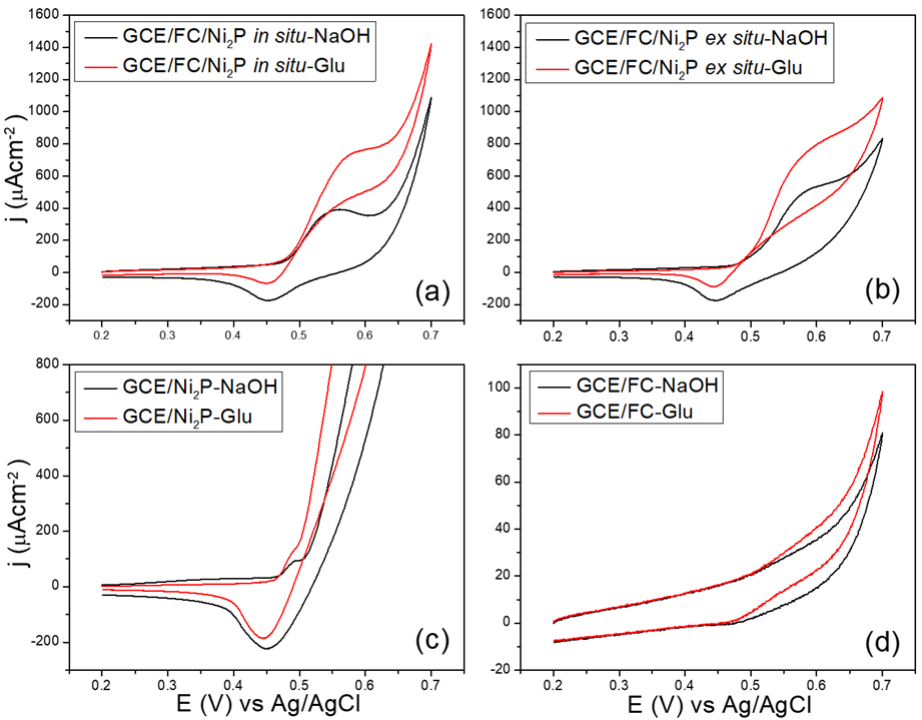
Cyclic voltammetry of (**a**) GCE/FC/Ni_2_P in situ, (**b**) GCE/FC/Ni_2_P ex situ, (**c**) GCE/Ni_2_P, and (**d**) GCE/FC modified electrodes in the presence and absence of glucose. Conditions: additions of 1.00 mM glucose, potential range from 0.200 to 0.700 V, scan rate = 50.0 mVs^−1^, 0.100 M NaOH.

The electrocatalytic behavior of modified glassy carbon electrodes was analyzed using cyclic voltammetry in the presence and absence of 1.00 mM glucose, using a potential range of 0.200 to 0.700 V in 0.100 M NaOH solution. The voltammograms obtained using the GCE/FC/Ni_2_P in situ, GCE/FC/Ni_2_P ex situ and GCE/Ni_2_P modified electrodes (Figure 3a–c, respectively) show that in the absence of glucose (black lines), a redox process occurs close to 0.450–0.500 V. According to the literature, we assign this process to the nickel Ni^2+^/Ni^3+^ redox process [9,10]. On the other hand, when the GCE/FC electrode is used no current response is observed in the potential range analyzed (Figure 3d). Moreover, when the pH was changed to 10 and 11 no signals were observed using either composite (Figure S6 for GCE/FC/Ni_2_P in situ electrode). In an alkaline medium the formation of the NiO_x_/Ni(OH)_x_ species on the Ni_2_P surface has been demonstrated, so it is reasonable to conclude that the observed peaks are derived from the surface faradaic reaction between the NiO_x_/Ni(OH)_x_ species and NiOOH [23], according to:

NiO_x_/Ni(OH)_x_ + OH^−1^ → NiOOH + e^−^ (1)

In the presence of 1.00 mM glucose, a remarkable increase in anodic current density is observed for the GCE/FC/Ni_2_P in situ electrode followed by the GCE/FC/Ni_2_P ex situ electrode, indicating that glucose electrooxidation was catalyzed effectively by these materials. In the case of the GCE/Ni_2_P electrode, the presence of glucose causes a small increase in the current density, showing that the presence of carbon fiber in the sensor improves the catalytic activity presented by Ni_2_P. As mentioned above, once the electrode is in a basic medium the formation of Ni(OH)_x_ and NiOOH species is possible on the Ni_2_P surface. Thus, when the applied oxidation potential is close to 0.500 V, NiO_x_/Ni(OH)_x_ species are electrochemically oxidized to NiOOH, which oxidizes glucose to gluconolactone [26,27]. The oxidation of glucose due to the electrocatalytic activity of NiOOH can be expressed by the following formulation:

NiOOH + glucose → NiO_x_/Ni(OH)_x_ + H_2_O + gluconolactone + e^−^ (2)

Therefore, the electro-oxidation of glucose is dependent on the formation of hydroxide species on the surface of the electrode and therefore on the pH in this system.

From the cyclic voltammetry study it is possible to point out that the in situ GCE/FC/Ni_2_P electrode provides a greater electrocatalytic effect for the oxidation of glucose under the conditions studied. The characteristics of this composite provide a greater synergistic effect between Ni_2_P and the carbon fiber. A greater interaction between its components, as well as a completely homogeneous distribution of Ni_2_P nanoparticles throughout the carbon material, is the main descriptor.

The influence of the scan rate on the electrochemical behavior was studied in the presence of glucose (1.00 mM). As shown in Figure S7a, the current intensities increase progressively with the scan rate for the 1.00 mM glucose solutions. A linear relationship between the logarithmic plot of current vs. scan rate is observed. The slope with a value of 0.600 indicates that the glucose oxidation process is mainly diffusion controlled. This is confirmed from the linear fit (R^2^ = 0.990) between the current density and the square root of the scan rate (Figure S7b).

### 3.3. Electroanalytical Studies

**Figure 4:**
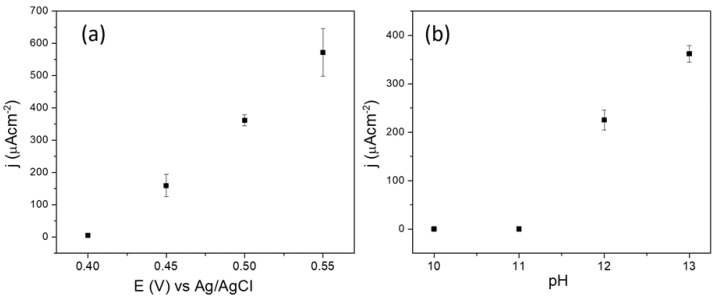
(**a**) Hydrodynamic voltammetry in 0.100 M NaOH solution and (**b**) effect of pH at 0.500 V for the GCE/FC/Ni_2_P in situ electrode in the presence of 300 µM glucose, corresponding to three measurements.

The first optimized parameters for obtaining the most sensitive responses to glucose detection were the applied potential and the pH medium. Figure 4a shows the hydrodynamic voltammogram in the presence of 300 µM glucose in 0.100 M NaOH in a range of potentials between 0.400 V and 0.550 V obtained with the GCE/FC/Ni_2_P in situ modified electrode. It can be seen that at 0.500 V, a considerably high current density concomitant with the smallest standard deviation was obtained. Additionally, the background current for 0.500 V was very low (data not shown) in comparison with the background for 0.550 V. In this way, a potential of 0.500 V was chosen as the optimal working potential for glucose detection in the following experiments.

The working pH is an important factor in the glucose oxidation reaction, so the electrochemical response of GCE/FC/Ni_2_P in situ after the addition of 300 µM glucose was investigated over a range of pH values from 10 to 13. From Figure 4b, it is observed that the anodic current density increases from pH 12 to 13 and is practically null at lower pH values. To corroborate that no electrochemical signal is obtained by amperometry at low pH values, cyclic voltammetry was performed over a wide range of potentials (Figure S3). The results show no signals related to glucose oxidation, proving the necessity of a highly basic environment for the occurrence of redox processes. On the other hand, the oxygen evolution reaction has been observed at higher pH values (pH ≈ 14) when metal phosphide catalysts are used [28]. Considering this, pH 13 was chosen as the optimal value for this study.

**Figure 5:**
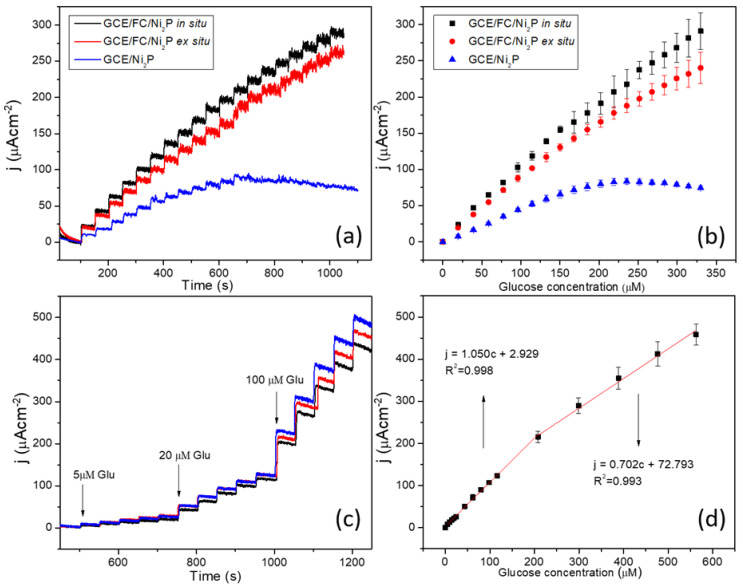
(**a**) Amperometric response of modified electrodes to successive addition of glucose in 0.100 M NaOH solution with a potential of 0.500 V vs. Ag/AgCl electrode and (**b**) current response as a function of glucose concentration, corresponding to three measurements. (**c**) Amperometric response of the GCE/FC/Ni_2_P in situ sensor to successive addition of glucose in 0.100 M NaOH solution with a potential of 0.500 V vs. Ag/AgCl electrode and (**d**) response in the current density as a function of glucose concentration, corresponding to three measurements.

Using 0.500 V in a 0.100 M NaOH solution, the comparative amperometric response of GCE/FC/Ni_2_P in situ, GCE/FC/Ni_2_P ex situ, and GCE/Ni_2_P electrodes was studied (Figure 5a). All electrodes present a fast amperometric response toward glucose sensing, reaching a stable current density in less than 5 s. The comparative analysis clearly shows that the GCE/FC/Ni_2_P in situ electrode has the most sensitive response against glucose electrodetection. An excellent linear relationship in the range of 19.8–150 µM (Figure 5b) was obtained, reaching a sensitivity of 1005 µAmM^−1^cm^−2^ (R^2^ = 0.998). In addition, with this sensor, the lowest detection and quantification limits (taken as 3.3 σ/S for detection limit and 10 σ/S for quantification limit, where σ is the standard deviation of the blank signal and S the sensitivity), as indicated in Table 1, were obtained.

A clear synergistic effect between the carbon fiber and Ni_2_P nanoparticles is observed, and both Ni_2_P/FC composites, in situ and ex situ, reach higher sensitivities than the GCE/Ni_2_P electrode. Considering that both composites have the same Ni_2_P:FC weight ratio (4:6), the homogeneous distribution of Ni_2_P nanoparticles onto the carbon fiber in the in situ route of synthesis should explain the greater effect observed. Finally, the linear response as a function of glucose concentration for the GCE/Ni_2_P sensor is more limited, exhibiting a dramatic loss of response after multiple glucose injections, which can be attributed to the saturation of the active sites.

Exploring the response of the GCE/FC/Ni_2_P in situ sensor with the addition of different concentrations of glucose, it can be observed from Figure 5c,d that the sensor has a linear response even at concentrations as low as 5 µM and continues responding to additions of 20 and 200 µM. The calibration curve of this sensor (Figure 5d) shows two concentration ranges with an excellent linear relationship between the current response and the glucose concentration. The sensor showed a sensitivity of 1050 µAmM^−1^cm^−2^ with a linear range of 5–208 µM (R^2^ = 0.998) and a response time of 2 s. The detection and quantification limits were 0.25 µM and 0.76 µM, respectively. At higher concentrations (208–563 µM), there is still a linear but less sensitive response (702 µAmM^−1^cm^−2^).

### 3.4. Sensor Stability and Interfering Studies

**Figure 6:**
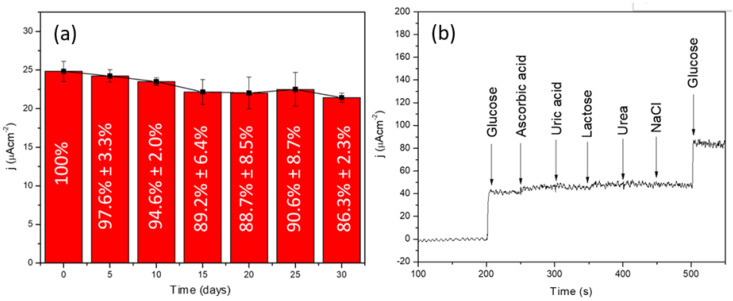
(**a**) Current response using the GCE/FC/Ni_2_P in situ sensor in the detection of 20.00 µM glucose in 0.100 M NaOH in consecutive measurements every 5 days for 30 days (corresponding to three measurements). (**b**) Amperometric responses of the GCE/FC/Ni_2_P in situ sensor to the successive addition of 100 µM glucose and 10 µM interferents.

The long-term stability of the sensor was also studied for practical applications. The GCE/FC/Ni_2_P in situ electrode was stored at room temperature, and the electrochemical response toward glucose was determined every 5 days for up to 30 days of storage. As shown in Figure 6a, the sensor retains 89.2% ± 6.4% of its initial current density after 15 days of storage. Furthermore, there is only a 13.7% decrease in its initial current density after 30 days of storage under ambient conditions, indicating high long-term stability.

Interference in the glucose oxidation response by other molecules present in the matrix to be analyzed is one of the main challenges in glucose sensing. In general, according to the literature, the normal glucose level in humans is more than ten times higher than that of common interferents [3]. The amperometric responses of the GCE/FC/Ni_2_P in situ electrode to the addition of 100 µM glucose before and after the addition of 10 µM ascorbic acid (AA), uric acid (AU), lactose, urea, and NaCl were evaluated. Figure 6b shows that the sensor has a high response for glucose with and without interferents, and there is no response toward interfering species. Therefore, it is verified that the GCE/FC/Ni_2_P in situ sensor is highly selective toward glucose concentrations.

### 3.5. Glucose Determination in Artificial Human Saliva

To assess the feasibility of the GCE/FC/Ni_2_P in situ sensor for glucose analysis in real samples, its accuracy in artificial human saliva (SAGF medium) was evaluated through amperometry recovery tests. A salivary glucose concentration of 150 µM corresponds to normal glucose levels in nondiabetic patients [29,30,31].

The samples were diluted in 0.100 M NaOH, resulting in final glucose concentrations of 18.0 µM in the cell at pH 13. Figure S8a represents the corresponding amperometric responses of the sensor. The first addition corresponds to the glucose sample in artificial saliva, followed by four standard additions of 100 µM glucose at the aforementioned glucose concentration. The current densities increase with the successive addition of glucose. The recovery percentage was 135.8% ± 1.8% of the theoretical glucose concentration levels tested. Although the recovery values are higher than 100%, the low error values obtained demonstrate the good precision and feasibility of the proposed sensor for the non-enzymatic determination of glucose in human saliva.

A performance comparison of the GCE/FC/Ni_2_P in situ electrode with other glucose sensors based on different nickel compounds, metal phosphides, and carbon fibers is summarized in Table 2.

Regarding glucose sensors based on Ni_2_P specifically [21,23], our work reports similar analytical performance but differs in several aspects such as synthesis of the composite (FC/Ni_2_P) in situ, nature of the carbon material, precursor of the carbon material, morphology and distribution of Ni_2_P nanoparticles on the carbon matrix, and applicability of the sensor in the detection of glucose on an artificial saliva matrix. Concerning the different nickel compounds, Ni(OH)_2_ and NiO have been extensively investigated for sensor applications. Ni(OH)_2_-based sensors usually involve potentials higher than ours, such as those reported by Guo et al. [32] and Yue Zhang et al. [33]. In addition, the latter, being analogous to our work due to the use of glassy carbon electrodes, rGO/Ni(OH)_2_/GCE, presents lower sensitivity and a higher detection limit compared to our results. On the other hand, NiO-based sensors, such as MWCNT/ NiO [34] and CPE/MWCNT/NiO [35], present considerably higher detection limits (2.0 μM and 19.0 μM, respectively). Compared to the different metal phosphides used in glucose sensors, Ni_5_P_4_/GCE [36] and Co_2_P/NPCNT/GCE [37], our sensor has higher sensitivity and lower detection limit. Another aspect important to remark is that our sensor requires a lower working potential compared to Ni_2_P-Cu_3_P/GCE [38] and Co_2_P/NPCNT/GCE sensors [37]. This comparison demonstrates the potential of nickel phosphide as an electrocatalytic material for glucose detection compared to other species of metal phosphides such as Ni_5_P_4_ and Co_2_P, as well as mixtures of Ni_2_P with oxides or other species of metal phosphides. Finally, the Table 2 also includes composites based on carbon fibers with different nickel species such as: Ni [39,40,41,42], NiCo_2_O_4_ [43,44], Ni(OH)_2_ [45] and CuO/NiO [46]. The comparison demonstrates that our sensor presents higher sensitivity compared to most of the works presented, except for the work of L. Liu et al. [43], however, this work uses a higher potential.

In addition, except for the work by Mei et al. [13], our sensor presents lower detection limits than these works.

**Table 2:** Sensing performance comparison of GCE/FC/Ni_2_P in situ with other non-enzymatic glucose sensors.

Sensor	Sensitivity (µAmM^−1^cm^−2^)	Detection Limit (µM)	Linear Range (µM)	Work Potential (V)	Real Sample	Ref.
GCE/FC/Ni_2_P in situ	1050	0.25	5–208	0.5	Artificial saliva	This work
GCE /Graphene/Ni_2_P(MOF)	7234	0.44	5–1400	0.5	Human serum	[22]
Carbon clothes/Ni_2_P Nanoarray	7792	0.18	1–3000	0.5	Human blood serum sample peach juice, and human blood	[21]
Nickel Foam/Ni_2_P	6375.1	0.14	2–937	0.5	Human serum	[23]
Ni_2_P/NiO/CeO_2_/Ni foam	28,230	0.5	1–250	0.5	Human serum	[47]
GCE/Ni_2_P-Cu_3_P	4700	0.1	4–5000	0.64	-	[38]
GCE/Ni_5_P_4_	149.6	0.7	2–5300	0.44	Human serum	[36]
GCE/NPCNT/Co_2_P	338.8	0.88	2000–7000	0.55	-	[37]
Ni(OH)_2_/PU PU = polyurethane	2845	0.32	10–2060	0.6	Human serum	[32]
GCE/RGO/Ni(OH)_2_	11.43	600	2–3100	0.6	-	[33]
MWCNT/NiO	1768.8	2	10–7000	0.5	Human blood	[34]
CPE/MWCNT/NiO	6527	19	1–14,000	0.5	Human blood	[35]
FTO/NiO	2632.53	0.084	5–825	0.55	human saliva	[13]
Ni/CFP	420.4	1	2–2500	0.6	-	[39]
Ni/CNF mat Carbon nanofiber	-	0.57	2–5000	0.55	Human serum	[40]
Ni-MOF/RGO/CF	852	0.6	6–2090	0.62	Orange juice	[41]
GCE/Ni-CoO/CNF	-	0.03	0.25–600	0.5	Human serum	[42]
ECF/NiCo_2_O_4_	1947.2	1.5	5–19,175	0.55	Human serum	[43]
0.5 Ni/ECNF-5 h	610.6	730	2000–10,000	0.5	Human serum	[44]
NiO/ECNF	557.68	850		0.5		
Co_3_O_4_/ECNF	475.72	820		0.6		
NiCo_2_O_4_/ECNF	536.5	930		0.55		
GCE /Ni(OH)_2_/CNF	1038.6	0.76	1000–1200	0.45	Fruit juice and Human serum	[45]
ACF/CuO/NiO	247	0.146	0.25–5000	0.55	Human serum	[46]

## 4. Conclusions

In summary, the synthetic route proposed in our work allows obtaining carbon fibers from the polyvinylpyrrolidone polymer (PVP) phosphoric acid and nickel sulphate, which allowed us to develop a reproducible nanomaterial using simple and cheap reagents compared to those methods that prepare graphene, or carbon cloth used in the aforementioned works. Electroanalytical experiments show the synergy between carbon fiber and Ni_2_P nanoparticles, with both composites, ex situ and in situ, being more electrocatalytic for glucose oxidation than Ni_2_P alone. Comparing both composites, the electrocatalytic performance of the in situ composite is better than that of the ex situ composite. Thus, we can conclude that the homogeneous distribution of Ni_2_P nanoparticles obtained under the in situ route guarantees a more sensitive glucose detection compared with the ex situ material. The GCE/FC/Ni_2_P in situ electrode is sensitive and selective toward glucose detection without interference from classical normal molecules present in physiological fluids. Regarding the applicability of the sensor in a real sample, our work demonstrates for the first time the detection of glucose in an artificial saliva matrix using a non-enzymatic sensor based on Ni_2_P. This has not been demonstrated for another metal phosphide-based sensor to date. Finally, it is clear that even when Ni compounds have been used for glucose sensors and that there are three works that use Ni phosphides, our sensor represents a novel and competitive alternative for the development of non-enzymatic glucose sensors.

## Figures and Tables

**Table 1:** Comparison of the analytical parameters of different modified electrodes.

Sensor	Sensitivity µAmM^−1^cm^−2^	LOD µM	LOQ µM	Concentration Range/µM
GCE/FC/Ni_2_P in situ	1005	0.24	0.73	19.8–185
GCE/FC/Ni_2_P ex situ	818	1.24 µM	3.75	19.8–185
GCE/Ni_2_P	424	1.34 µM	4.07	19.8–185

## Data Availability

Not applicable.

## Supplementary Materials

The following supporting information can be downloaded at: https://www.mdpi.com/article/10.3390/bios13010049/s1, Figure S1: Scanning transmission electron microscopy (STEM) of the (a) in situ FC/Ni_2_P composite and (c) Ni_2_P. (b) Gaussian particle size distribution of the in situ FC/Ni_2_P composite and (d) Ni_2_P from STEM images. Figure S2: SEM images of fibers obtained by electrospinning (a) without heat treatment, (b) dried at 110 °C, (c) stabilized at 250 °C, and (d) carbonized at 700 °C, with their average diameters. Figure S3: SEM images of fibers obtained by electrospinning (a) without heat treatment, (b) dried at 110 °C, (c) stabilized at 250 °C, and (d) carbonized/reduction at 700 °C, with their average diameters. Figure S4: EDX spectrum per point of Figure 2, corresponding to FC/Ni_2_P in situ composite. Table S1: Summary EDX analysis by point of the in situ FC/Ni_2_P composite of Figure 2. Figure S5: Thermogravimetric analysis (TGA) of in situ FC/ Ni_2_P composite. Figure S6: Cyclic voltammetry of GCE/FC/Ni_2_P in situ modified electrodes in the presence (red lines) and absence of glucose (black lines) in different concentration of NaOH solution (a) pH = 10 (b) pH = 11 and (c) pH = 12. Conditions: additions of 1.00 mM glucose, scan rate= 50.0 mVs-1. Figure S7: (a) Cyclic voltammetry of the GCE/FC/Ni_2_P in situ electrode in the presence of 1mM glucose at different scan rates (mVs^−1^). (b) the corresponding plots of current density vs. the square root of scan rate. Figure S8: Amperometric response of glucose in artificial saliva for standard addition method (a) 18 µM and (b) correspond to the calibration curves, corresponding to three measurements.
