# Supplementary material for: Synergistic Effect of Composite Nickel Phosphide Nanoparticles and Carbon Fiber on the Enhancement of Salivary Enzyme-Free Glucose Sensing
Source: Biosensors (Basel). 2022 Dec 29;13(1):49. doi: 10.3390/bios13010049 (PMC9856003; doi:10.3390/bios13010049)
Supplement: Supplementary file 1 [file biosensors-13-00049-s001.zip › biosensors-2065598-supplementary.pdf]

biosensors

MDPI

Supporting Information

Synergistic Effect of Composite Nickel Phosphide Nanoparticles and Carbon Fiber on the Enhancement of Salivary Enzyme-Free Glucose Sensing

Tania P. Brito [1,2,3], Nicole Butto-Miranda [4], Andrónico Neira-Carrillo [4], Soledad Bollo [1,*] and Domingo Ruíz-León [2,*]

[1] Centro de Investigación de Procesos Redox (CiPRex), Facultad de Ciencias Químicas y Farmacéuticas, Universidad de Chile, Santiago 8330015, Chile
[2] Laboratorio de Fisicoquímica y Electroquímica del estado Sólido, Facultad de Química y Biología, Universidad de Santiago de Chile, Santiago 8330015, Chile
[3] Departamento de Ingeniería Metalúrgica, Facultad de Ingeniería, Universidad de Santiago de Chile, Santiago 8330015, Chile
[4] Departamento de Ciencias Biológicas Animales, Facultad de Ciencias Veterinarias y Pecuarias, Universidad de Chile, Santiago 8330015, Chile
* Correspondence: sbollo@ciq.uchile.cl (S.B.); domingo.ruiz@usach.cl (D.R.-L.)

Figure S1: Scanning transmission electron microscopy (STEM) of the (a) *in situ* FC/Ni_2P composite and (c) Ni_2P. (b) Gaussian particle size distribution of the *in situ* FC/Ni_2P composite and (d) Ni_2P from STEM images.

Figure S2: SEM images of fibers obtained by electrospinning (a) without heat treatment, (b) dried at 110°C, (c) stabilized at 250°C, and (d) carbonized at 700°C, with their average diameters.

Figure S3: SEM images of fibers obtained by electrospinning (a) without heat treatment, (b) dried at 110°C, (c) stabilized at 250°C, and (d) carbonized/reduction at 700°C, with their average diameters.

Figure S4: EDX spectrum per point of Figure 2, corresponding to FC/Ni_2P *in situ* composite.

Table S1: Summary EDX analysis by point of the *in situ* FC/Ni_2P composite of figure 2.

Figure S5: Thermogravimetric analysis (TGA) of *in situ* FC/ Ni_2P composite.

Figure S6: Cyclic voltammetry of GCE/FC/Ni_2P *in situ* modified electrodes in the presence (red lines) and absence of glucose (black lines) in different concentration of NaOH solution (a) pH = 10 (b) pH = 11 and (c) pH = 12. Conditions: additions of 1.00 mM glucose, scan rate= 50.0 mVs^{-1}.

Figure S7: (a) Cyclic voltammetry of the GCE/FC/Ni_2P *in situ* electrode in the presence of 1mM glucose at different scan rates (mVs^{-1}). (b) the corresponding plots of current density vs the square root of scan rate.

Figure S8: Amperometric response of glucose in artificial saliva for standard addition method (a) 18 μM and (b) correspond to the calibration curves, corresponding to three measurements.

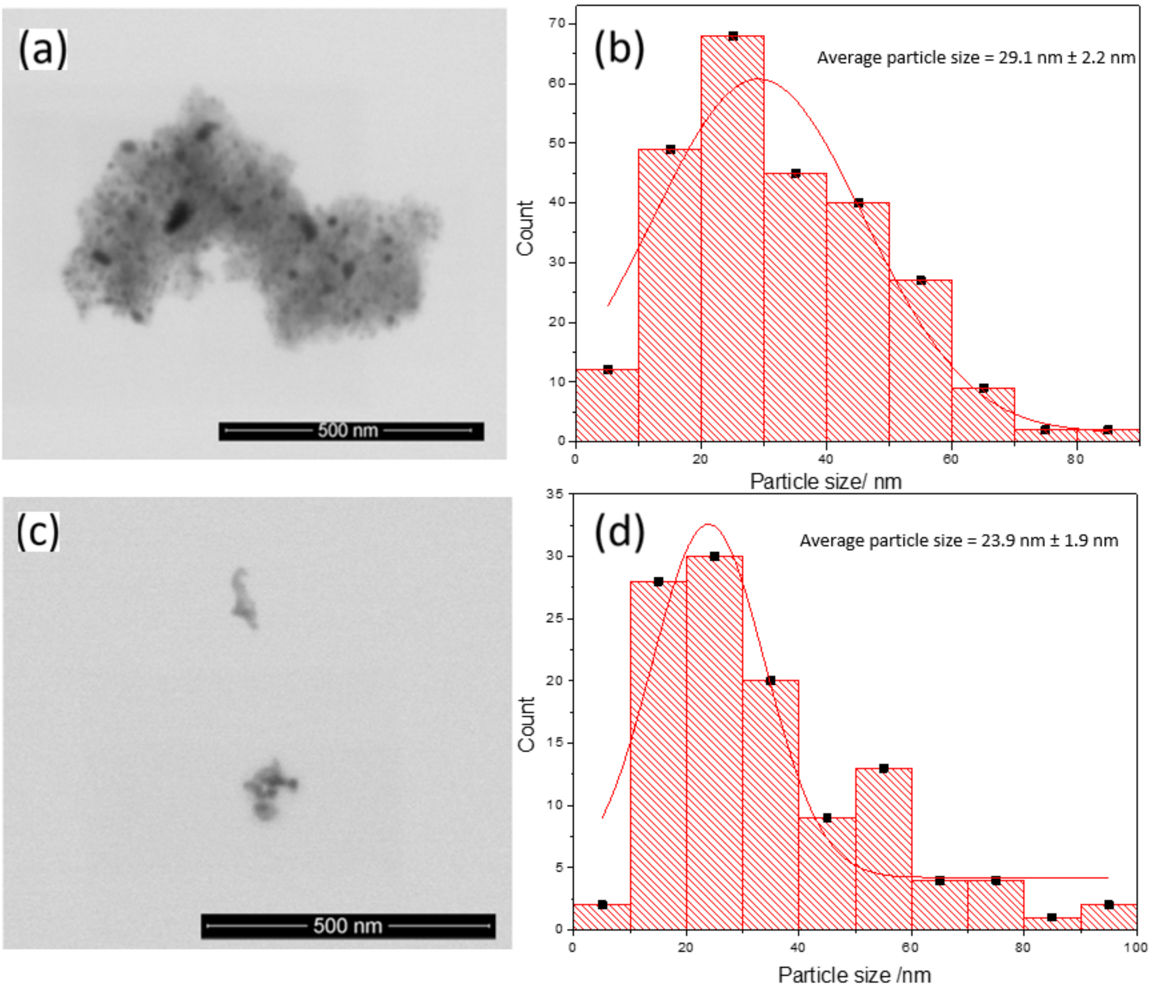

Figure S1. Scanning transmission electron microscopy (STEM) of the (**a**) *in situ* FC/Ni_2P composite and (**c**) Ni_2P. (**b**) Gaussian particle size distribution of the *in situ* FC/Ni_2P composite and (**d**) Ni_2P from STEM images.

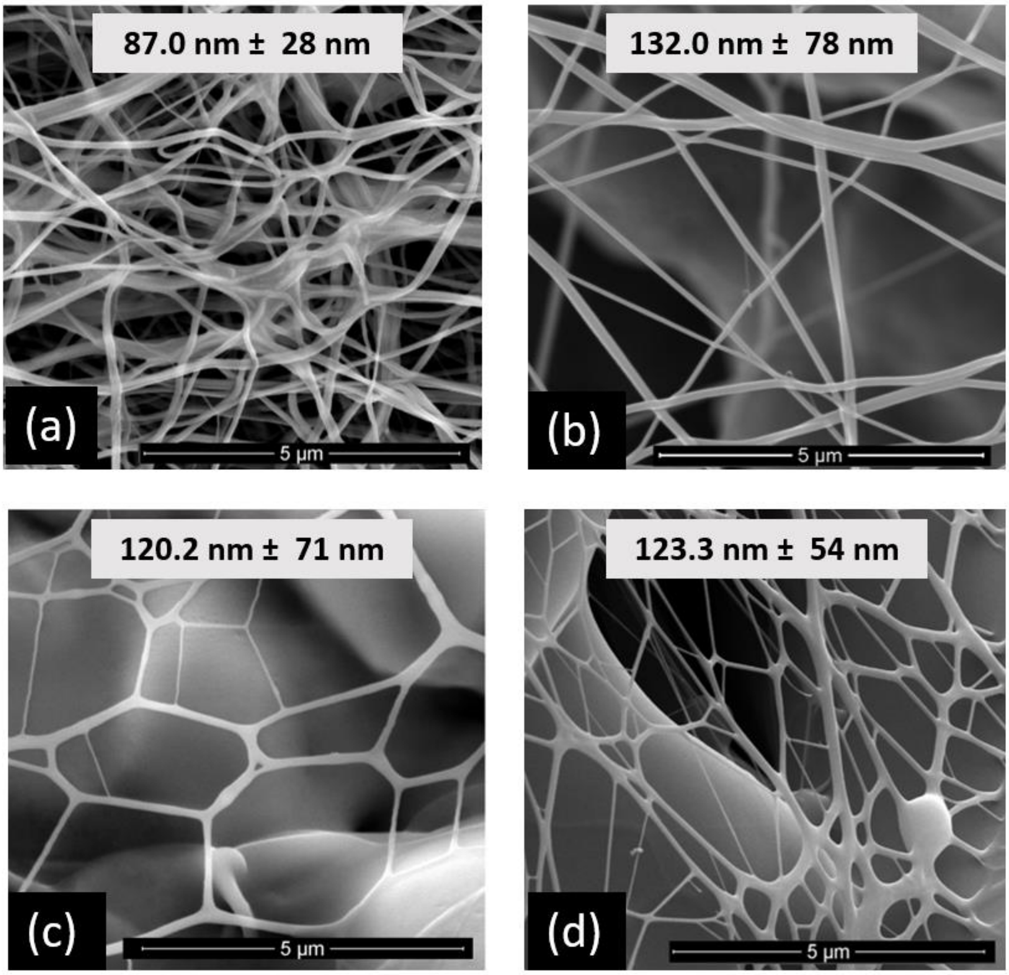

Figure S2. SEM images of fibers obtained by electrospinning (a) without heat treatment, (b) dried at 110°C, (c) stabilized at 250°C, and (d) carbonized at 700°C, with their average diameters.

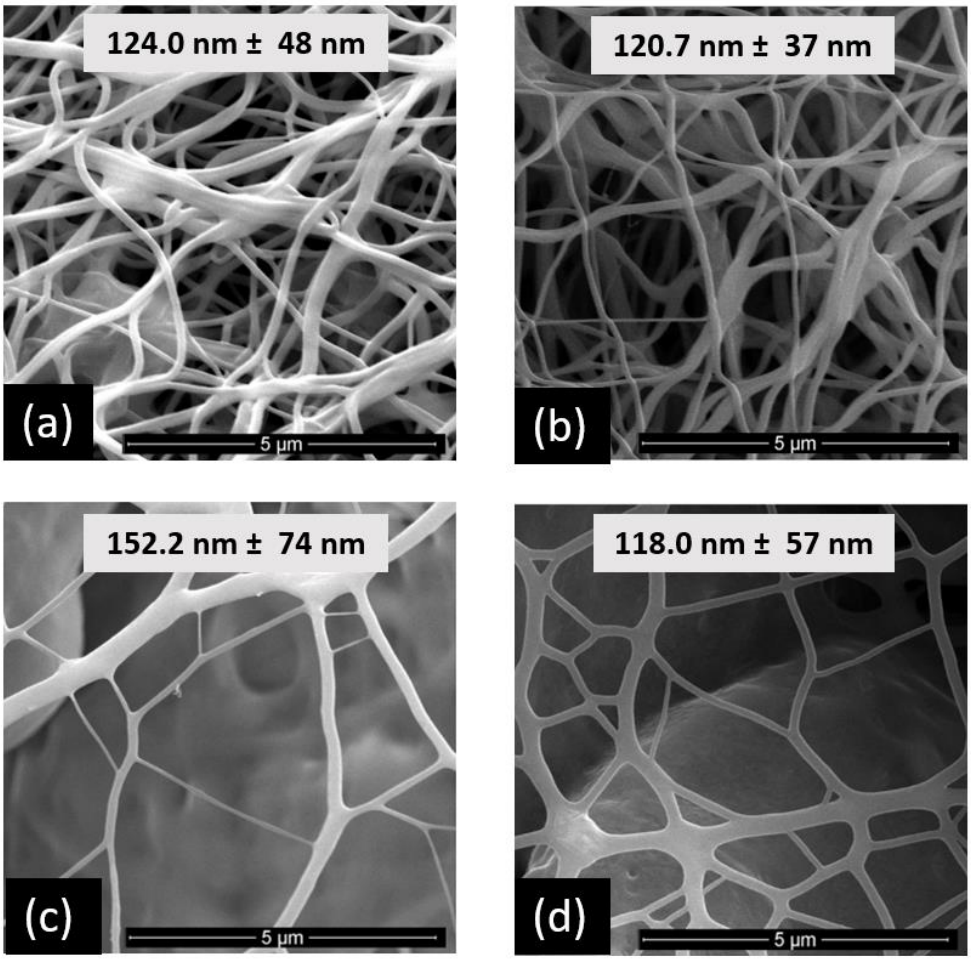

Figure S3. SEM images of fibers obtained by electrospinning (a) without heat treatment, (b) dried at 110°C, (c) stabilized at 250°C, and (d) carbonized/reduction at 700°C, with their average diameters.

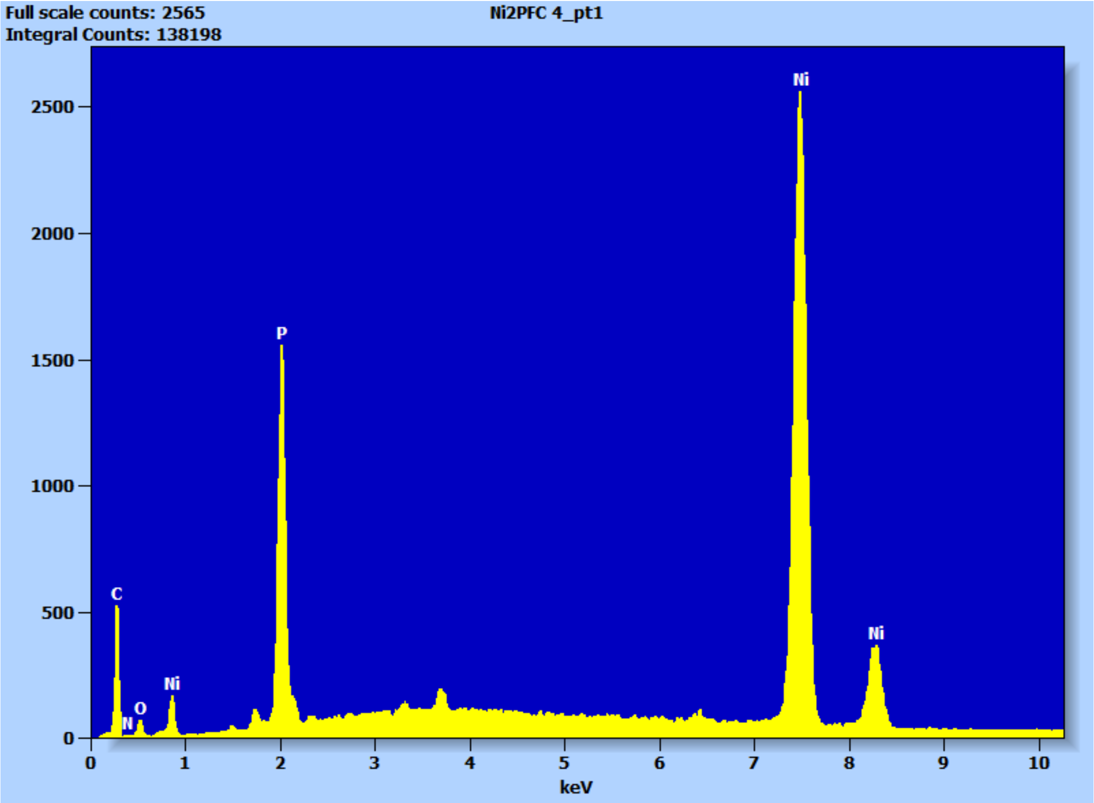

Figure S4. EDX spectrum per point of Figure 2, corresponding to FC/Ni_2P in situ composite.

Table S1: Summary EDX analysis by point of the in situ FC/Ni2P composite of figure 2.

Element	Point 1	Point 2	Point 3	Point 4	Point 5
	Atom %	Atom %	Atom %	Atom %	Atom %
P K	31.58	31.90	31.98	31.62	32.26
Ni K	68.42	68.10	68.02	68.38	67.74
	100.00	100.00	100.00	100.00	100.00

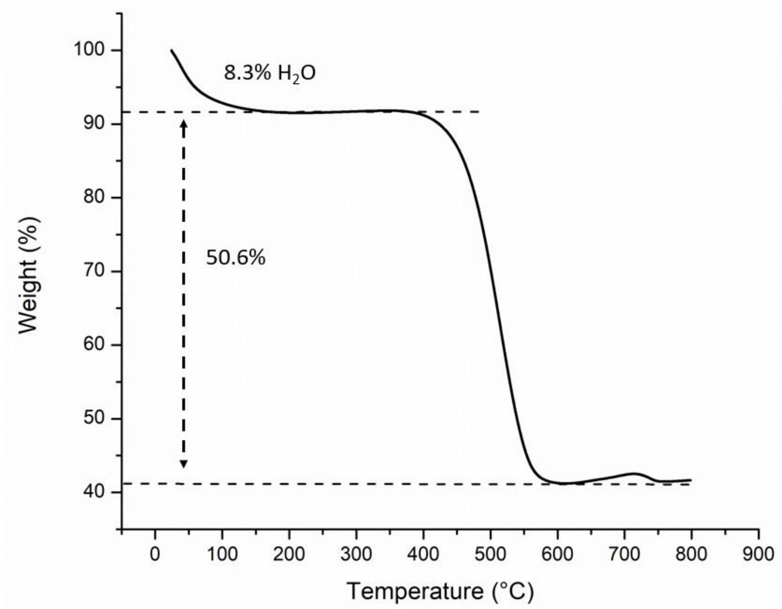

Figure S5. Thermogravimetric analysis (TGA) of *in situ* FC/ Ni_2P composite.

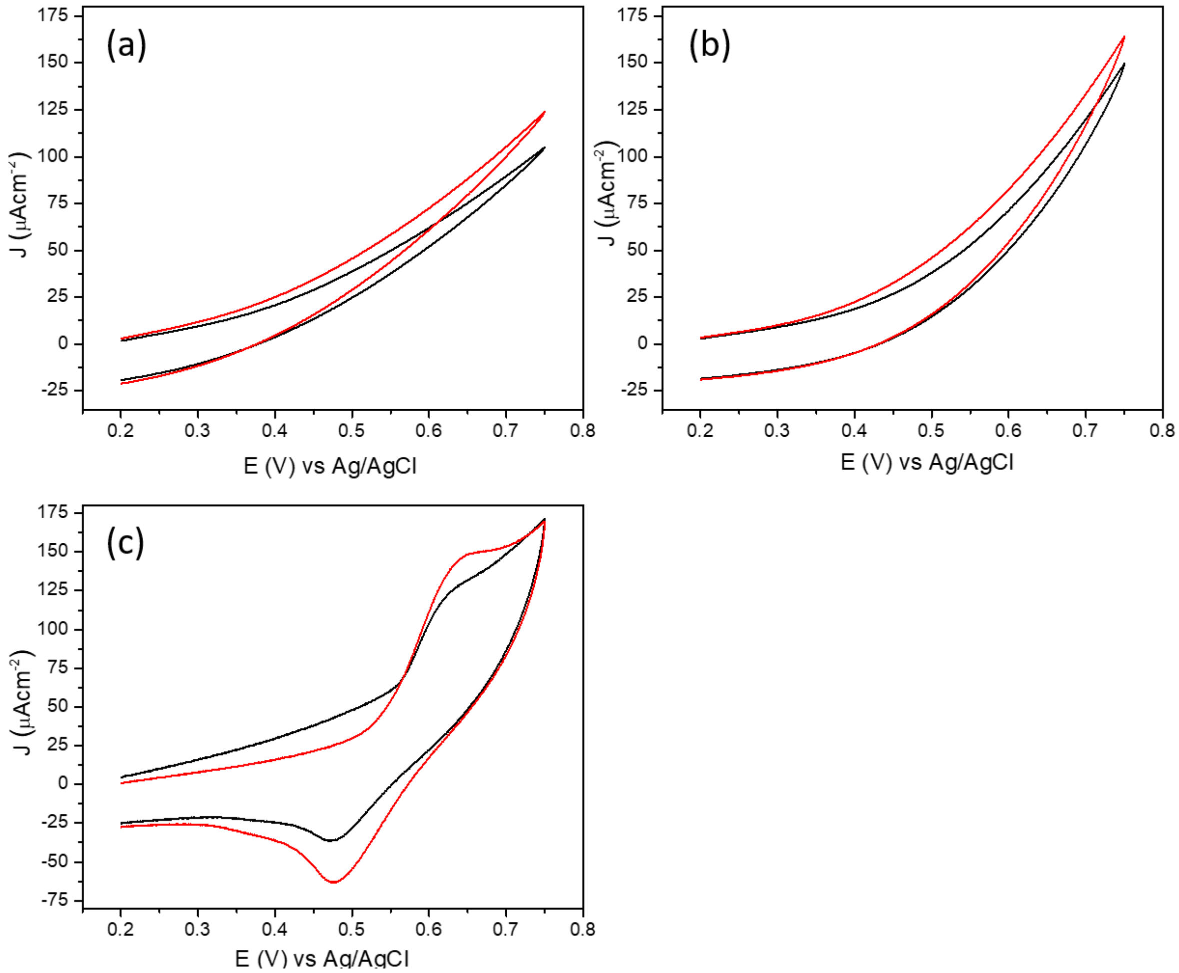

Figure S6. Cyclic voltammetry of GCE/FC/Ni_2P *in situ* modified electrodes in the presence (red lines) and absence of glucose (black lines) in different concentration of NaOH solution (**a**) pH = 10 (**b**) pH = 11 and (**c**) pH = 12. Conditions: additions of 1.00 mM glucose, scan rate= 50.0 mVs^{-1}.

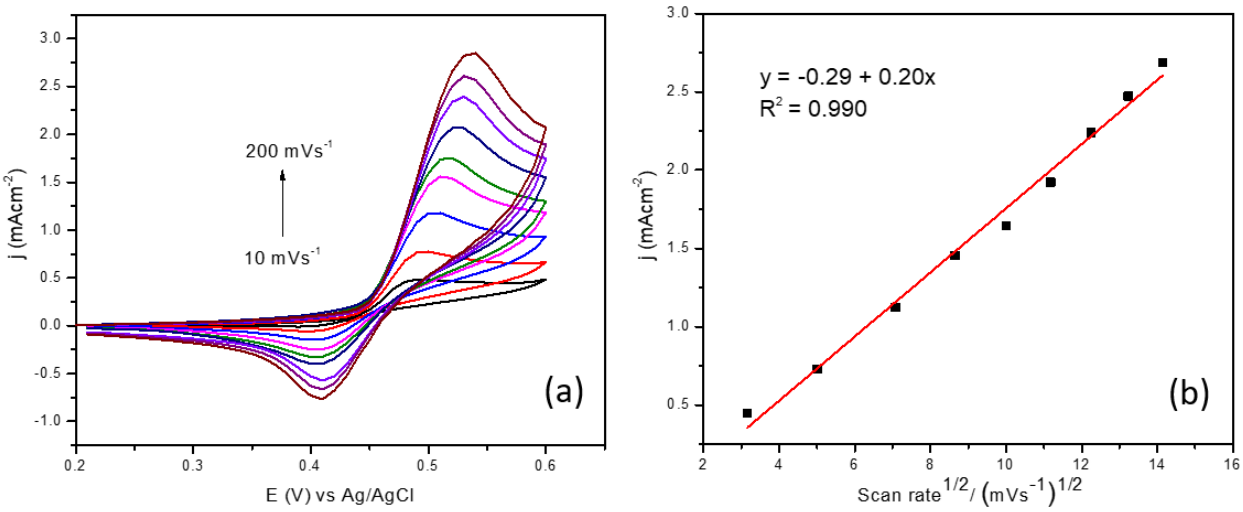

Figure S7. (**a**) Cyclic voltammetry of the GCE/FC/Ni_2P *in situ* electrode in the presence of 1mM glucose at different scan rates (mVs^{-1}). (**b**) the corresponding plots of current density vs the square root of scan rate.

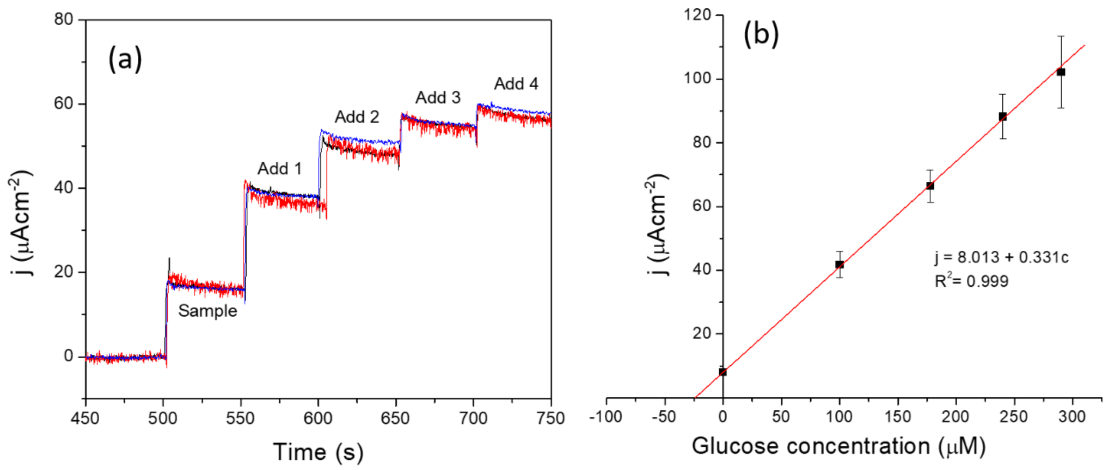

Figure S8. Amperometric response of glucose in artificial saliva for standard addition method (**a**) 18 μM and (**b**) correspond to the calibration curves, corresponding to three measurements.
